# Severe Pneumonia Caused by Coinfection With Influenza Virus Followed by Methicillin-Resistant *Staphylococcus aureus* Induces Higher Mortality in Mice

**DOI:** 10.3389/fimmu.2018.03189

**Published:** 2019-01-30

**Authors:** Leili Jia, Jiangyun Zhao, Chaojie Yang, Yuan Liang, Pengwei Long, Xiao Liu, Shaofu Qiu, Ligui Wang, Jing Xie, Hao Li, Hongbo Liu, Weiguang Guo, Shan Wang, Peng Li, Binghua Zhu, Rongzhang Hao, Hui Ma, Yong Jiang, Hongbin Song

**Affiliations:** ^1^Academy of Military Medical Sciences, Academy of Military Sciences, Beijing, China; ^2^Chinese PLA Center for Disease Control and Prevention, Beijing, China; ^3^Department of Health Care, Chinese PLA Joint Staff Headquarters Guard Bureau, Beijing, China; ^4^305 Hospital of PLA, Beijing, China; ^5^The 6th Medical Center of Chinese PLA General Hospital, Beijing, China; ^6^Guangdong Provincial Key Laboratory of Proteomics, State Key Laboratory of Organ Failure Research, School of Basic Medical Sciences, Southern Medical University, Guangzhou, China

**Keywords:** coinfection, influenza, methicillin-resistant *Staphylococcus aureus*, bacteria, pneumonia

## Abstract

**Background:** Coinfection with influenza virus and bacteria is a major cause of high mortality during flu pandemics. Understanding the mechanisms behind such coinfections is of utmost importance both for the clinical treatment of influenza and the prevention and control of epidemics.

**Methods:** To investigate the cause of high mortality during flu pandemics, we performed coinfection experiments with H1N1 influenza virus and *Staphylococcus aureus* in which mice were infected with bacteria at time points ranging from 0 to 7 days after infection with influenza virus.

**Results:** The mortality rates of mice infected with bacteria were highest 0–3 days after infection with influenza virus; lung tissues extracted from these co-infected mice showed higher infiltrating cells and thicker lung parenchyma than lung samples from coinfected mice in which influenza virus was introduced at other times and sequences. The levels of interferon (IFN)-γ, tumor necrosis factor (TNF)-α, interleukin (IL)-8, and IL-6 in the 0–3 day coinfected group were significantly higher than those in the other groups (*p* < 0.01), as were the mRNA levels of IFN-γ, IL-6, and TNF-α. Coinfection with influenza virus and *S. aureus* led to high mortality rates that are directly dependent on the sequence and timing of infection by both pathogens. Moreover, coinfection following this particular schedule induced severe pneumonia, leading to increased mortality.

**Conclusions:** Our data suggest that prevention of bacterial co-infection in the early stage of influenza virus infection is critical to reducing the risk of clinical mortality.

## Introduction

Recent studies have shown that 50–90% of severe or fatal cases of influenza virus infection during previous influenza pandemics and seasonal epidemics have been associated with bacterial coinfection (1–4). It has also been reported that there are at least 15 types of bacteria that can infect individuals simultaneously with influenza virus. At least 4 types of these bacteria (*S. aureus, Streptococcus pneumonia, Legionella pneumophila*, and *Haemophilus influenzae*) increase the risk of pneumonia and subsequent mortality (5–8). Additionally, *Pseudomonas aeruginosa* was recently reported to cause severe pneumonia when coinfected with influenza (9). Some studies have shown that patients with *S. aureus* and influenza virus coinfection have the highest mortality rates (10, 11). During the H1N1 influenza pandemic of 2009, a study in the Netherlands revealed that *S. aureus* was a co-pathogen in 59% of confirmed influenza patients, which was ~4-fold higher than the rate of *S. pneumoniae* (15%) (12). A study in the UK found that *S. aureus* was detected in 27% of patients with confirmed influenza A at 140 hospitals, which was higher than the detection rates of *S. pneumoniae* and *Haemophilus* (15 and 4%, respectively) (13). However, the mechanism by which coinfection with influenza and *S. aureus* leads to higher mortality remains unclear. More importantly, methicillin-resistant *S. aureus* (MRSA), which has become prevalent in recent years (14), is resistant to almost all β-lactam antibiotics including penicillin and cephalosporins, making conventional antibiotic treatment difficult (9, 15). Vancomycin is generally considered to be effective against MRSA, although recently discovered strains are resistant to it as well (15). In this study, a high-mortality mouse model was established based on coinfection with influenza A (H1N1 A/Puerto Rico/8/34) and MRSA with the aim of revealing the activation status of pneumonia during coinfection as well as the mechanism by which it produces high mortality.

## Materials and Methods

### Mice

Six-week-old C57Bl/6 (B6) female mice were purchased from the Laboratory Animal Center of the Academy of Military Medical Sciences of China. All mice were housed under specific pathogen-free conditions at the Laboratory Animal Center of the Academy of Military Medical Sciences of China. All experiments were performed in accordance with the relevant institutional animal care and use guidelines.

### Viral Strains and Bacteria

The A/Puerto Rico/8/34 (H1N1) strain of influenza A virus (PR8) was maintained in this laboratory and replicated in Madin-Darby Canine Kidney cells for later use (16). MRSA was isolated from the sputum of a patient with necrotizing pneumonia and initially stored in our laboratory (−80°C), after which it was grown in Luria-Bertani medium (Solarbio, Beijing, China) at 37°C to the stationary phase for subsequent experiments.

### Infections and Groups

Mice were anesthetized via injection with 2% pentobarbital sodium (40 μL/10 g body weight) and then infected intranasally with influenza virus and bacteria. A quantity of 10^0.5^ (~3.162) influenza viruses at a 50% tissue culture infective dose/mL was adjusted to 25 μL with phosphate-buffered saline (PBS), and a MRSA quantity of 5 × 10^7^ colony-forming units/mL was adjusted to 40 μL with PBS. Body weights and any deaths in mice were determined daily. The mice were grouped as shown in Table 1.

**Table 1:** Group descriptions.

**Groups**	**Description**
d+1	Secondary inoculation 1 day after MRSA
d+2	Secondary inoculation 2 day after MRSA
d+3	Secondary inoculation 3 day after MRSA
d-0	Secondary inoculation 0 day after IAV
d-1	Secondary inoculation 1 day after IAV
d-2	Secondary inoculation 2 day after IAV
d-3	Secondary inoculation 3 day after IAV
d-4	Secondary inoculation 4 day after IAV
d-5	Secondary inoculation 5 day after IAV
d-6	Secondary inoculation 6 day after IAV
d-7	Secondary inoculation 7 day after IAV

*“d” denotes day. MRSA, methicillin-resistant S. aureus; IAV, influenza A virus*.

### Hematoxylin and Eosin Staining

Fresh lung tissues were fixed in 4% paraformaldehyde for over 24 h. The lung tissues were then embedded with paraffin and sliced into 3–5 μm-thick sections. Pathological scores were evaluated in a blinded randomized manner. The specific evaluation indicators are as follows: 0 = no airway necrosis, 1 = focal necrosis, 2 = some confluence of necrosis in larger airways, and 3 = confluent airway necrosis in most airways.

### Quantitative PCR for Determining Viral and Bacterial Loads

Mouse lungs were removed post-necropsy and placed in 2 mL EP tubes, after which 1 mL of PBS and 1% protease inhibitor (CW Biotech, Beijing, China) were added and the EP tubes were automatically ground in a high-throughput tissue grinder. The supernatant was collected after centrifugation at 10,000 g in 4°C for 15 min. The influenza virus titer in the lungs was measured using the fluorescence quantitative PCR method. The plate count method was used for quantitative determination of *S. aureus*. The lungs were triturated with a Polytron PT100 (Glen Mills, Clifton, NJ, USA) and the volume was adjusted to 1 mL with PBS to achieve the desired viral and bacterial titers.

### ELISA for Measurement of Cytokines Expression Levels

Bronchoalveolar lavages were performed as previously reported (16). Briefly, the mice were anesthetized with pentobarbital sodium, and the fat and connective tissue surrounding the trachea were dissected to sufficiently expose the trachea. PBS was aspirated with a 1 mL syringe equipped with a blunt 12-gauge needle; 5 mL was slowly injected along the trachea into the bronchi, and this bronchoalveolar lavage fluid (BALF) was slowly aspirated 1 min later. The supernatant was separated via centrifugation at 1,300 rpm, and the concentrations of cytokines in the mouse alveolar lavage fluid, serum, and lung homogenates were detected using mouse IFN-γ, TNF-α, IL-6, and IL-8 ELISA kits (Jymbio,Wuhan,China) according to the manufacturers' instructions.

### Measurement of Cytokine mRNA Using PCR

Trizol (Invitrogen, Carlsbad, CA) reagent was used to extract total RNA from the lung tissue of infected mice according to the manufacturer's instructions. Each RNA sample is treated with DNase I (Fermentas, Harrington, Canada) to eliminate DNA contamination. cDNA was reverse-transcribed from 6 μg of RNA using SMART® MMLV Reverse Transcriptase (TaKaRa, Dalian, Japan). Quantitative real-time PCR was performed with EvaGreen qPCR Master Mix in a LightCycler® 480II system (Roche, Basel, Switzerland) to measure the gene expression of IFN-γ, TNF-α, IL-6, and IL-8. The following primers were used for amplification: mouse IFN-γ, 5′ -GACATGAAAATCCTGCAGAGC- 3′ and 5′-TGAGCTCATTGAATGCTTGG- 3′; mouse TNF-α, 5′-TAACTTAGAAAGGGGATTATGGCT-3′ and 5′-TGGAAAGGTCTGAAGGTAGGAA-3′; mouse IL-6, 5′-TGATGCACTTGCAGAAAACA-3′ and 5′-GGTCTTGGTCCTTAGCCACTC-3′; mouse IL-8, 5′ -GGTCTTCCTGCTTGAATGGCTTGA-3′ and 5′ -GCGGTGTCCTGATTATCGTCCTC-3′; mouse β-actin, 5′ -TCCATCATGAAGTGTGACGT- 3′ and 5′ -GAGCAATGATCTTGATCTTCA−3′. Relative target expression was calculated according to the 2^−Δ*ΔCt*^ method of Livak and Schmittgen and expressed as the fold change of the different genes compared with the housekeeping gene β*-actin*.

### Statistical Analysis

Statistical analyses of the experimental results were performed using 2-way *t*-tests, and the experimental data are presented as mean ± standard error of mean: *p* < 0.05 indicates a significant difference.

## Results

### Mortality Rates in Mice Are Dependent on the Sequence and Timing of Infection With the 2 Pathogens

**Figure 1:**
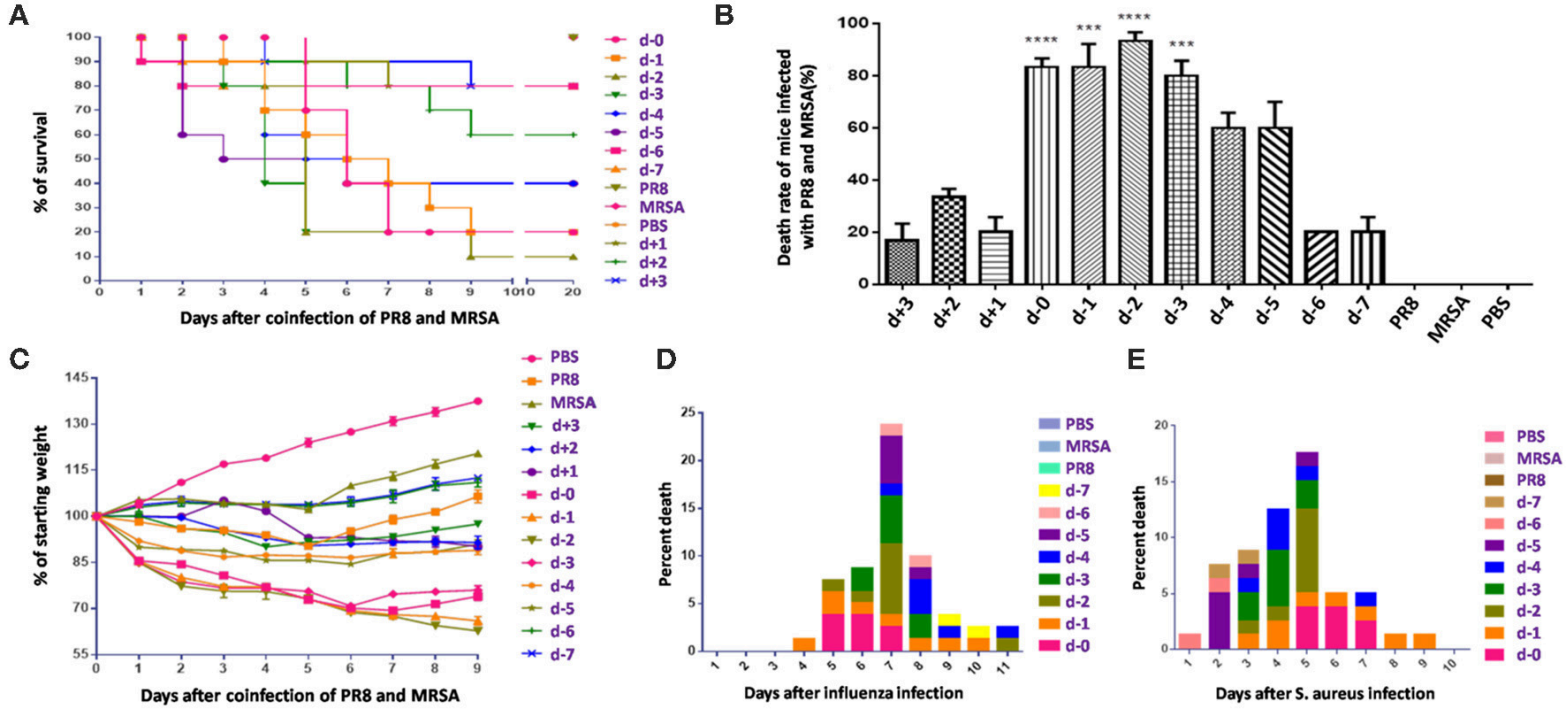
Mortality in mice coinfected with influenza virus and methicillin-resistant *S. aureus* differed with the sequence and timing of infection. **(A)** Survival rates in mice coinfected with influenza virus (PR8) and methicillin-resistant *S. aureus* (MRSA) were infection sequence- and time-dependent. In mice coinfected with equivalent doses, those first infected with PR8 followed by MRSA had higher mortality rates than those first infected with MRSA. **(B)** Mortality of mice coinfected using various infection sequences and timings (***p < 0.001; ****p < 0.0001, *n* = 6). **(C)** Body weight changes in coinfected mice. **(D)** The cumulative mortality rate after coinfection of mice in groups d to d-7 with PR8 shows that the coinfected mice had the highest mortality rate on the seventh day post-PR8 infection. **(E)** The cumulative mortality rate after groups d to d-7 were coinfected with MRSA; mortality rates rose in the first 5 days after MRSA infection.

We first used the mouse model to explore coinfection-induced death. To minimize the impact of the pathogens themselves on coinfection-related mortality, various coinfection sequences and time points were planned using non-lethal doses of influenza virus (PR8) and *S. aureus* (MRSA). The results (Figures 1A,B,D,E) indicated that mortality is significantly related to the sequence and timing of infection with both pathogens. Specifically, mortality was highest (>80%) in mice infected with MRSA [d, d-1, d-2, and d-3 groups (Table 1)] from day 0 to day 3 post-infection with influenza virus, which was significantly higher than that in other coinfected groups. In particular, the mortality of mice infected with bacteria first followed by influenza virus, was ≤ 40%. Although the mortality rates in the d-4 and d-5 groups also increased, these were relatively lower (~60%). The mortality rates in the d-6, d-7, d+1, d+2, and d+3 groups were under 40%; those in the d-6, d-7, d+1, and d+3 groups were ≤20%, while the survival rates in the PR8-, MRSA-, and PBS-only groups were 100%. The body weights of mice exhibiting high mortality showed a marked downward trend (Figure 1C).

### Histopathology and Distribution of Influenza Virus and MRSA in the Lung Differ According to Coinfection Conditions

**Figure 2:**
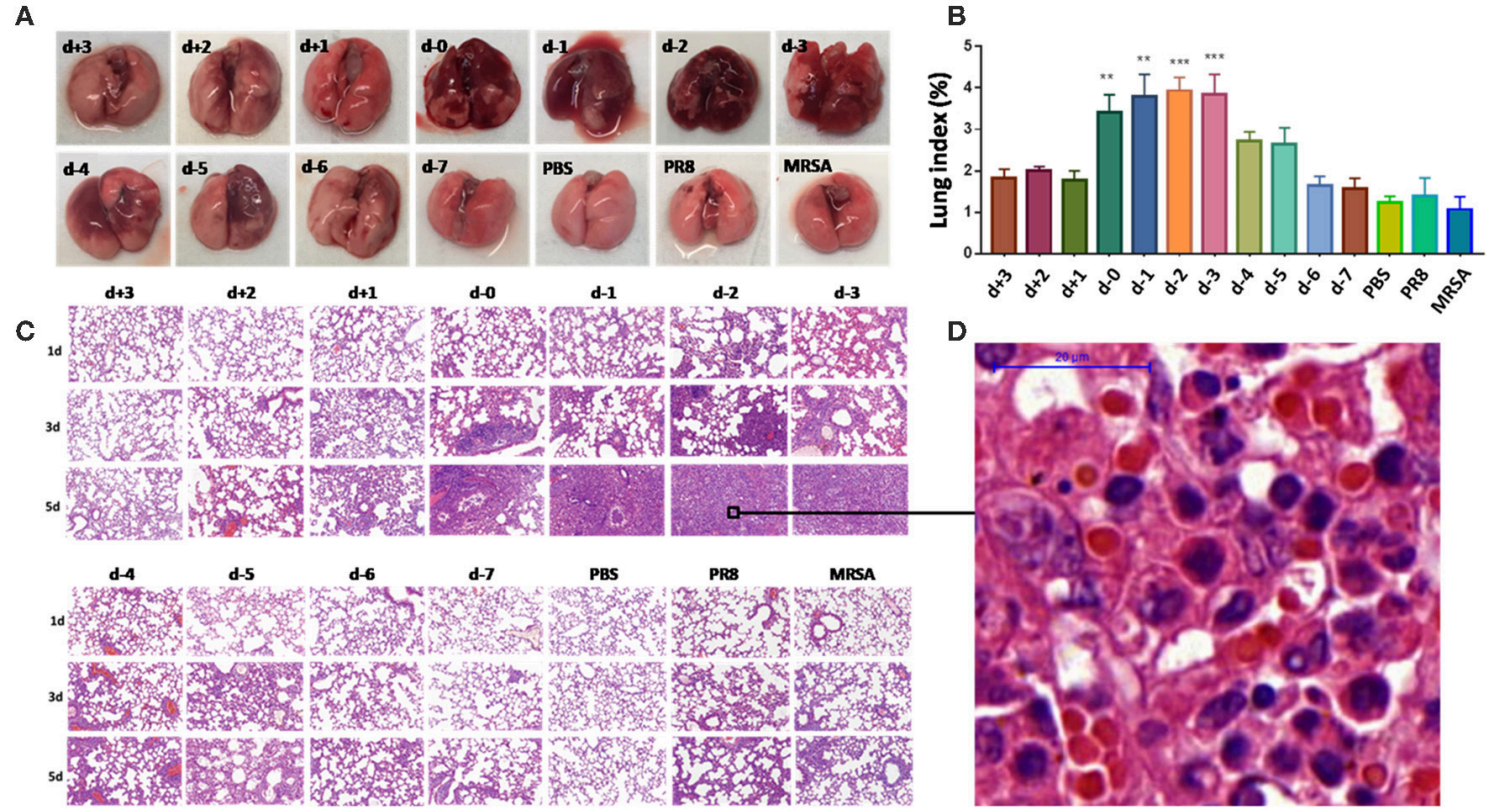
Pathological differences in mice coinfected with influenza virus and methicillin-resistant *S. aureus* using different infection sequences and times. **(A)** Anatomy of the lung tissues of coinfected mice with different infection sequences and times. Lung tissue injury in groups d-0, d-1, d-2, and d-3 was significantly more severe than that in the other groups as denoted by the color change from pink to dark purple. **(B)** The lung indices of mice in groups d-0, d-1, d-2, and d-3 were significantly higher than in the other groups (**p < 0.01; ***p < 0.001, *n* = 6). **(C)** Lung pathological sections of coinfected mice with different infection sequences and times (15 × ). Lung injury in groups d-0, d-1, d-2, and d-3 on the fifth day after infection was significantly higher than in the other groups. **(D)** Local enlarged view of pathological sections of lungs showing severe lung with 100 × magnification. The alveoli showed extensive atrophy or did not dilate. Pulmonary consolidation, in which severe infiltration of inflammatory cells (lymphocytes and granulocytes), was observed. PR8, influenza A virus strain; MRSA, methicillin-resistant *S. aureus*; PBS, phosphate-buffered saline.

**Figure 3:**
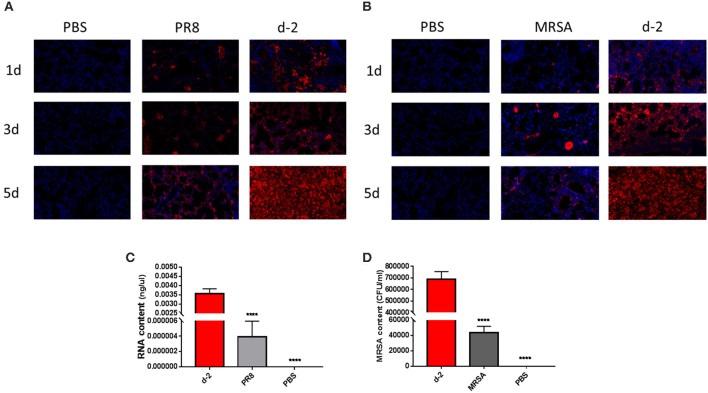
Over-proliferation of pulmonary pathogens in coinfected mice in group d-2. **(A)** PR8 in the lungs of group d-2 mice showed significant proliferation between day 1 and day 5 after coinfection compared to the control PR8 group and the group infected with a non-lethal dose of PR8 alone. **(B)** MRSA in the lungs of group d-2 mice showed significant proliferation between day 1 and day 5 after coinfection compared with the control PR8 group and the group infected with a non-lethal dose of MRSA alone. **(C)** Real-time fluorescence quantitative PCR was used to detect the mRNA levels of PR8 in lung homogenates from group d-2 mice acquired on the fifth day of coinfection. Compared with the control PBS group and the group infected with a non-lethal dose of PR8 alone, mRNA levels in group d-2 far exceeded those in the control groups (****p < 0.0001, *n* = 6). **(D)** Lung homogenate from mice in the d-2 group was obtained on the fifth day post-coinfection, and bacterial culture was performed. Compared with the control PBS group and the group infected with a non-lethal dose of PR8 alone, the MRSA bacterial load in the lungs of group d-2 mice far exceeded those of the control groups (****p < 0.0001, *n* = 6). PR8, influenza A virus strain; MRSA, methicillin-resistant *S. aureus*; PBS, phosphate-buffered saline.

Lungs from coinfected mice that had high mortality exhibited markedly increased evidence of injury (Figures 2A–D). Lung injury areas and lung indices in mice infected with *S. aureus* (d, d-1, d-2, and d-3 groups) between day 0 and day 3 post-influenza virus infection were significantly higher than those in the other groups, and were accompanied by significant pulmonary parenchymal disease and infiltration of inflammatory cells (lymphocytes and granulocytes), alveolar atrophy, or consolidation. Pathological sections revealed severe lung damage as well as extensively atrophied or non-dilated alveoli. Meanwhile, analysis of the distribution of PR8 and MRSA in the lung during the course of the disease in coinfected mice with high mortality suggested that both pathogens produced a significant increase in lung load over time compared to the control group (Figure 3).

### Inflammatory Factor Levels in Lung BALF Were Elevated in Mice With Higher Mortality

**Figure 4:**
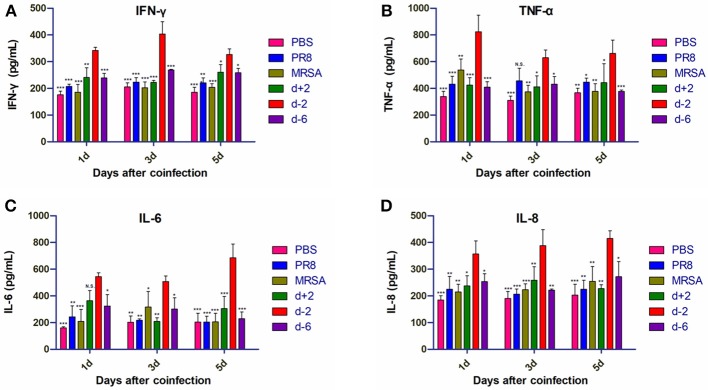
Inflammatory factor levels in the BALF of mice coinfected with influenza virus and methicillin-resistant *S. aureus*. Levels of **(A)** IFN-γ, **(B)** TNF-α, **(C)** IL-6, and **(D)** IL-8 in the BALF of coinfected mice are shown at different infection sequences and times. For multiple comparisons, we used one-way analysis of variance with the Tukey *post hoc* test. Data are represented as mean ± standard error of the mean. (N.S., not significant; *p < 0.05; **p < 0.01; ***p < 0.001, *n* = 6). BALF, bronchioalveolar lavage fluid; PR8, influenza A virus strain; MRSA, methicillin-resistant *S. aureus*; PBS, phosphate-buffered saline. IFN, interferon; TNF, tumor necrosis factor; IL, interleukin.

Levels of IFN-γ and IL-8 in the lung BALF of mice in group d-2 were significantly higher than in the other groups on the first, third, and fifth days post-coinfection (Figure 4). Tumor necrosis factor (TNF)-α levels in group d-2 were not significantly different from those in the PR8 group on the third day post-coinfection, but were significantly higher than the other groups on the first, third, and fifth days post-coinfection. Interleukin (IL)-6 levels in group d-2 were not significantly different than in the d+2 group on the first day after coinfection, but were significantly higher than in the other groups on the first, third and fifth days thereafter.

### mRNA Levels of Inflammatory Factors Differed Based on the Timing of Coinfection

**Figure 5:**
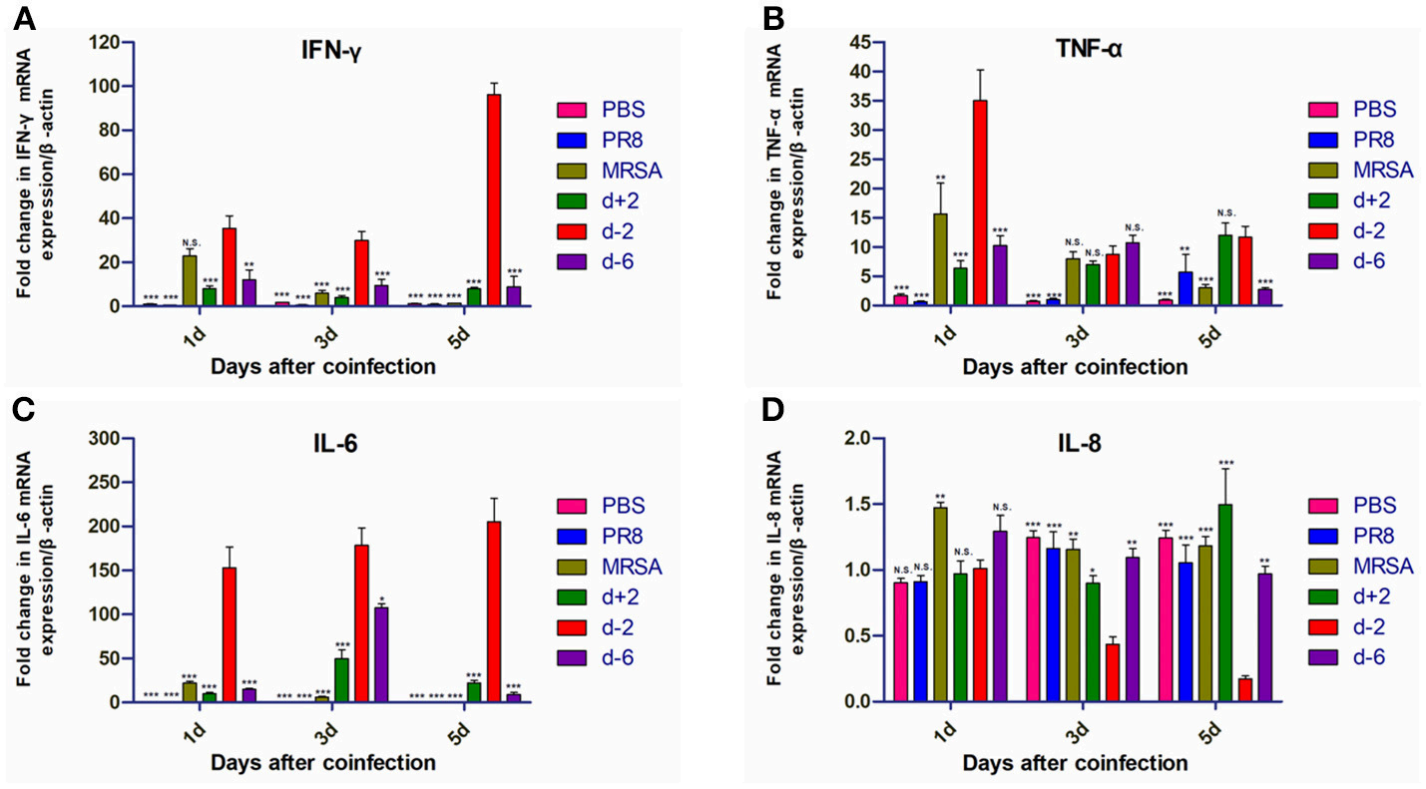
Inflammatory cytokine mRNA levels in mice coinfected with influenza virus and methicillin-resistant *S. aureus*. mRNA levels of **(A)** IFN-γ, **(B)** TNF-α, **(C)** IL-6, and **(D)** IL-8 in the lung tissues of coinfected mice according to different infection sequences and times. For multiple comparisons, we used one-way analysis of variance with the Tukey *post hoc* test. Data are represented as mean ± standard error of the mean. (N.S., not significant; *p < 0.05; **p < 0.01; ***p < 0.001, *n* = 6). PR8, influenza A virus strain; MRSA, methicillin-resistant *S. aureus*; PBS, phosphate-buffered saline. IFN, interferon; TNF, tumor necrosis factor; IL, interleukin.

The mRNA levels of interferon (IFN)-γ, TNF-α, and IL-6 in the lung tissues of mice in group d-2 were significantly higher than in the other groups on the first, third, and fifth days after coinfection (Figure 5). IFN-γ mRNA levels in group d-2 did not significantly differ from those in the MRSA group on the first day after coinfection, but were significantly higher than the other groups on the third and fifth days thereafter. mRNA levels of TNF-α were higher than in the other groups on the first day after coinfection, but were not significantly different from other co-infected groups on the third day after coinfection and were higher than the d-6 group on the fifth day after. mRNA levels of IL-6 in group d-2 were significantly higher than in the other groups on the first, third, and fifth days after coinfection, while mRNA levels of IL-8 in the lung tissues of the mice in group d-2 were not significantly different from the other groups on the first day but were lower on the third and fifth days post-coinfection.

## Discussion

Our results suggest that mortality owing to coinfection with influenza virus and *S. aureus* is related to the timing and sequence of infection of these 2 pathogens. Our most noteworthy finding was that high mortality in mice was associated with the development of severe pneumonia. The inflammatory characteristics not only manifested with the clear infiltration of inflammatory cells into the site of injury in the lung, but also by increased levels of these inflammatory factor protein and mRNA levels. These data point to severe pneumonia as playing a key role in the death of mice coinfected with influenza virus followed by *S. aureus*.

Investigators have described several models of pneumonia caused by influenza/MRSA coinfection (17–20). In our study, we found that coinfection-related mortality is linked to the order and timing of inoculation; i.e., mortality was highest (>80%) upon initial infection with influenza virus followed by infection with *S. aureus* between days 0 and 3. This phenomenon has also been reported in mice coinfected with influenza virus and *S. pneumoniae* (21), except that infection with *S. pneumoniae* on days 5–7 post-influenza virus infection still led to high mortality. This may be related to the type and dose of bacterial infection; for example, Jamieson et al. (7) demonstrated that mice infected with *Legionella pneumophila* on the third day after infection with influenza virus had the highest mortality (up to 100%). Our study revealed that PR8 and MRSA proliferated at a significantly higher rate than controls on the first day of coinfection (d-2), causing severe pneumonia symptoms (Figure 3). Other studies also observed pathogen proliferation following coinfection (22, 23).

Studies have shown that influenza virus co-infected with *S. aureus, S. pneumonia*, or *L. pneumophila* can cause more serious histopathological damage to lung tissues in mice (5–7, 17). In our study, the pathological changes induced by bacteria were most pronounced 0–3 days after the first infection with influenza virus. The pathological injury was most severe in the group infected with *S. aureus* on the second day after infection with influenza virus. In this experimental group (d-2), the lung tissues showed characteristics of severe damage, the alveoli were extensively atrophied or non-dilated, and the lung exhibited parenchymal disease by the third day post-coinfection. Extensive inflammatory cell infiltration (mainly of lymphocytes and granulocytes) was observed in the parenchymal lung tissue sections. Furthermore, the lung distribution of PR8 and MRSA during the course of coinfection increased gradually, which resulted in additional pulmonary damage.

Importantly, the most potent sequence of coinfection in our study induced a strong inflammatory response in the lungs that was similar to the pathological features observed in clinical patients post-infection (24). Namely, the levels of IFN-γ, TNF-α, IL-6, and IL-8 in the BALF of mice were all significantly increased in group d-2. Moreover, the mRNA levels of IFN-γ, TNF-α, and IL-6 also increased significantly in addition to those of IL-8. The most prominent changes were in IFN-γ and IL-6 protein and mRNA levels, which were elevated 1–5 days post-coinfection. IFN-γ can induce the expression of major histocompatibility complex class II antigens such as monocytes, macrophages, dendritic cells, dermal fibroblasts, vascular endothelial cells, and stellate cells, which participate in the process of immune recognition (25–28). Moreover, IFN-γ can promote the expression of FcγR in macrophages, synergistically induce TNF, and promote the destruction of pathogenic microorganisms by macrophages. In the present study, we found that the expression levels of IFN-γ increased in the PR8-infected group alone; this was also observed in previous studies (26, 27, 29). There was no increase in IFN-γ in the MRSA-infected group alone; however, the levels of IFN-γ expression in the co-infected group were significantly higher than those in the control group on days 1, 3, and 5 (Figure 4A). This result suggests that, although MRSA alone does not increase the expression levels of IFN-γ, it enhances influenza virus-induced increases in IFN-γ expression, thereby inducing a severe pneumonia response. In our study, IFN-γ levels in group d-2 were highest on the third day, while mRNA levels were highest on the fifth. Since changes in the remaining groups were not significant except in terms of bacterial infection, it appears that the observed phenotypic changes are mainly a consequence of *S. aureus* infection while the influenza virus served as an aggravator. IL-6 is a critical inflammatory cytokine (30) that is closely linked with the occurrence and development of infection; its protein levels are related to the severity of pneumonia and patient prognosis. High concentrations of IL-6 can directly damage endothelial cells, promote immune adhesion, form microthrombi, and inhibit endothelial repair, which in turn delays blood vessel damage and maintains high vascular permeability that can result in reduced immune tolerance. In our study, IL-6 protein and mRNA levels in the d-2 group were significantly increased, although changes among the other groups were not obvious. These results suggest that inflammatory factor levels can vary depending on the sequence of influenza and MRSA coinfection.

A limitation of this study was that it only investigated coinfection-related mortality as related to severe pneumonia, but did not clarify the mechanism of pneumonia development during coinfection; hence, additional research is required in that regard. However, our data suggest that preventing bacterial co-infection in the early stages of influenza virus infection is important for averting death.

## Ethics Statement

This study was carried out in accordance with the principles of the Basel Declaration and recommendations of Safety Committee of Chinese PLA Center for Disease Control and Prevention. The protocol was approved by the Safety Committee of Chinese PLA Center for Disease Control and Prevention.

## Author Contributions

HS, YJ, HM and LJ designed the research studies. JZ, YL, CY, XL, and PLo conducted experiments and acquired the data, while SQ, LW, JX, HaL, WG, RH, PLi, and BZ analyzed the data. HoL and SW provided reagents. HS, YJ, LJ, and YL wrote the manuscript.

### Conflict of Interest Statement

The authors declare that the research was conducted in the absence of any commercial or financial relationships that could be construed as a potential conflict of interest.
